# Multiplexed Guide RNA Expression Leads to Increased Mutation Frequency in Targeted Window Using a CRISPR-Guided Error-Prone DNA Polymerase in *Saccharomyces cerevisiae*

**DOI:** 10.1021/acssynbio.2c00689

**Published:** 2023-07-24

**Authors:** Michael Gossing, Angelo Limeta, Christos Skrekas, Mark Wigglesworth, Andrew Davis, Verena Siewers, Florian David

**Affiliations:** † Discovery Sciences, Biopharmaceuticals R&D, AstraZeneca, SE-41320 Gothenburg, Sweden; ‡ Department of Life Sciences, Chalmers University of Technology, SE-41296 Gothenburg, Sweden; § Novo Nordisk Foundation Center for Biosustainability, Technical University of Denmark, DK-2800 Kgs. Lyngby, Denmark; ∥ Discovery Sciences, Biopharmaceuticals R&D, AstraZeneca, Alderley Park SK10 2NA, U.K.; ⊥ Discovery Sciences, Biopharmaceutical R&D, AstraZeneca, Cambridge CB2 0AA, U.K.; # Alderley Lighthouse Laboratories Ltd., Alderley Park SK10 4TG, Macclesfield, U.K.

**Keywords:** *in vivo* mutagenesis, targeted mutagenesis, CRISPR, multiplexing, directed evolution, yeast

## Abstract

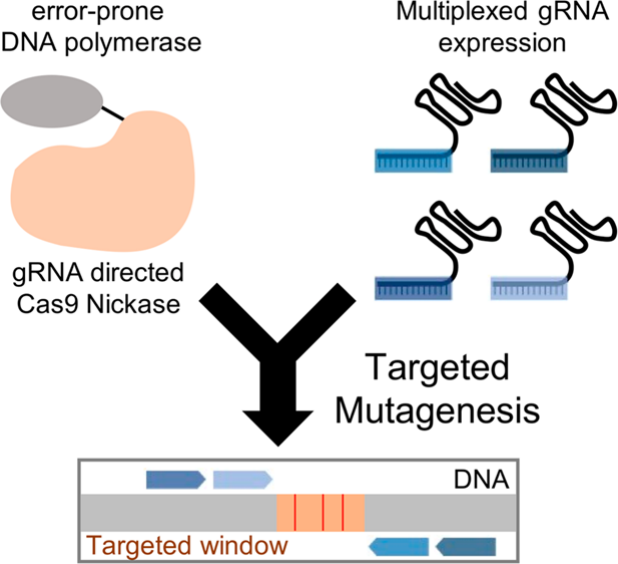

Clustered regularly interspaced short palindromic repeats (CRISPR)-Cas9 technology, with its ability to target a specific DNA locus using guide RNAs (gRNAs), is particularly suited for targeted mutagenesis. The targeted diversification of nucleotides in *Saccharomyces cerevisiae* using a CRISPR-guided error-prone DNA polymerase—called yEvolvR—was recently reported. Here, we investigate the effect of multiplexed expression of gRNAs flanking a short stretch of DNA on reversion and mutation frequencies using yEvolvR. Phenotypic assays demonstrate that higher reversion frequencies are observed when expressing multiple gRNAs simultaneously. Next generation sequencing reveals a synergistic effect of multiple gRNAs on mutation frequencies, which is more pronounced in a mutant with a partially defective DNA mismatch repair system. Additionally, we characterize a galactose-inducible yEvolvR, which enables temporal control of mutagenesis. This study demonstrates that multiplex expression of gRNAs and induction of mutagenesis greatly improves the capabilities of yEvolvR for generation of genetic libraries *in vivo*.

## Introduction

Directed evolution is a powerful method to optimize genetically encoded molecules toward a user-defined goal through iterative rounds of diversification, selection, and amplification. Targeted *in vivo* mutagenesis enables continuous diversification of a genetically encoded molecule in a cellular host. Several targeted mutagenesis methods that use the yeast *Saccharomyces cerevisiae* as a cellular host have been reported, using base editors guided by Cas9 variants or T7 RNA polymerase, orthogonal DNA replication systems, or retrotransposons.^1−4^

Recently, a method using an error-prone version of DNA Polymerase I guided by a Cas9 nickase, called yEvolvR, has been described.^5^ A Cas9 nickase contains inactivating mutations in either of its two endonuclease domains, resulting in the introduction of single-strand nicks instead of double-strand breaks into the target DNA sequence. After dissociation of the nickase, DNA Polymerase I binds and initiates strand displacement synthesis, followed by flap cleavage of the displaced original strand. This leaves a ligatable nick, which is repaired by endogenous DNA ligases. The error-prone nature of DNA synthesis performed by the here used DNA Polymerase I low-fidelity variant results in the introduction of mismatched base pairs. Mismatches that escape repair by endogenous DNA repair mechanisms, such as the Mismatch Mediated Repair (MMR) system, are ultimately converted to mutations.

**Figure 1:**
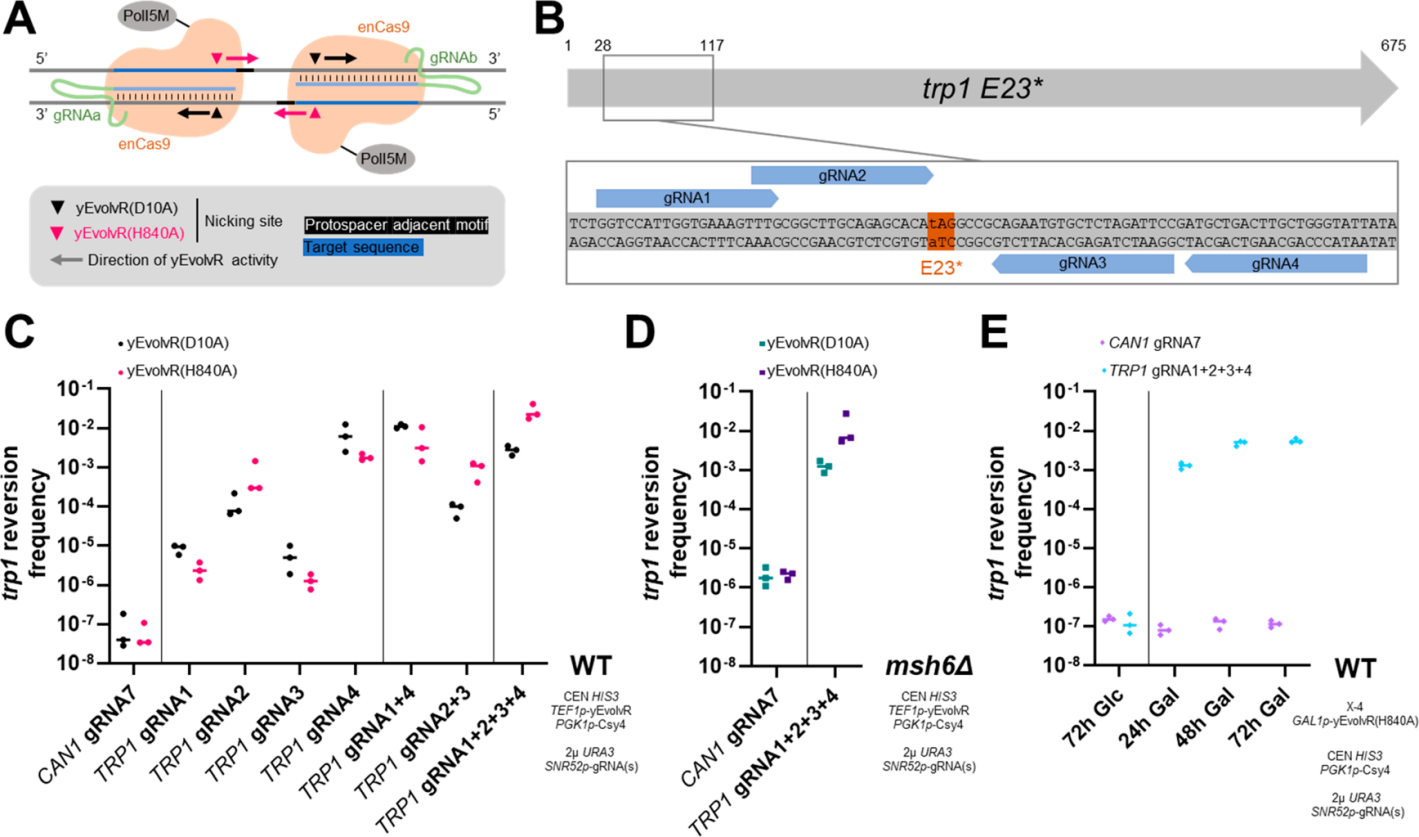
Multiplexed gRNA expression increases *trp1* reversion frequency with yEvolvR. (A) The direction of yEvolvR activity is dependent on the enCas9 nickase variant, and on which strand the target DNA sequence is located. (B) The introduced premature stop codon of *trp1* E23* is flanked by four gRNA target sequences, all within a ∼100 bp stretch of DNA. Numbers indicate the base pair number of the open reading frame. (C) Yeast cells were transformed with plasmids encoding yEvolvR, *csy4*, and gRNA(s), and liquid minimal medium containing tryptophan was directly inoculated with the transformants. After 72 h, *trp1* reversion frequencies were determined. (D) Yeast cells with a partially defective MMR system were transformed with plasmids encoding yEvolvR, *csy4*, and gRNA(s), and liquid minimal medium containing tryptophan was directly inoculated with the transformants. After 72 h, *trp1* reversion frequencies were determined. (E) Yeast cells with a genomically integrated copy of yEvolvR(H840A) under control of the galactose-inducible *GAL1p* promoter, which were previously transformed with plasmids encoding *csy4* and gRNA(s), were cultivated in liquid minimal medium containing tryptophan and the indicated carbon source (Glc, glucose; Gal, galactose). After the indicated amount of time (24, 48, or 72 h), *trp1* reversion frequencies were determined. Data points are biological triplicates. The bar indicates the median *trp1* reversion frequency.

The direction of yEvolvR activity is dependent on the Cas9 nickase variant, and on which strand the target sequence is located. Cas9 nickase D10A contains an inactivated RuvC endonuclease domain, resulting in nicking of the strand complementary to the gRNA. Cas9 nickase H840A contains an inactivated HNH endonuclease domain, resulting in nicking of the strand noncomplementary to the gRNA^6^ (Figure 1A). DNA Polymerase I then starts DNA synthesis from the resulting nick in the 5′–3′ direction. Mutation frequencies are increased in a ∼40 bp window in the expected direction of yEvolvR activity. Interestingly, in *S. cerevisiae*, an increase in mutation frequencies is also observed within a ∼15-bp window in the opposite direction of expected yEvolvR activity. The comparatively small mutational window of yEvolvR requires multiple gRNAs to enable mutagenesis of an open reading frame (ORF), with gRNAs spread out over the entirety of the sequence.^7^ Multiple gRNAs can also be used to target two ORFs simultaneously.^5^ Large and diverse genetic libraries are critical for successful directed evolution of a gene of interest. Increased mutation rates when using yEvolvR would facilitate generation of improved genetic libraries *in vivo*. We wanted to investigate if we could further improve mutation frequencies and expand the targeted window with yEvolvR by multiplexed gRNA expression, with all gRNAs flanking a small stretch of DNA.

## Results and Discussion

### Design of Mutagenesis System

The yEvolvR constructs used in this study consist of a fusion of either the D10A or H840A nickase variant of enhanced Cas9 (eCas9 v1.1) originating from *Streptococcus pyogenes*, and the DNA polymerase I quintuple mutant PolI5M originating from *Escherichia coli*, as described previously.^5,8,9^ We also investigated the role of *MSH6*, a component of the MMR system, whose gene product is involved in repair of single base pair mismatches.^10,11^ Additionally, we created a galactose-inducible yEvolvR system under control of a *GAL1p* promoter to enable temporal control of mutagenesis. Multiplexed gRNAs were generated from a single transcript by processing with the bacterial endonuclease Csy4 from *Pseudomonas aeruginosa*.^12^ yEvolvR and *csy4* were expressed from a centromeric plasmid under control of the *TEF1p* and *PGK1p* promoter, respectively. gRNAs were expressed under control of the RNA polymerase III promoter *SNR52p* and a *SUP4t* terminator from a 2 μ plasmid. High expression levels of gRNAs have been demonstrated to be beneficial for genome editing efficiency with Cas9.^13^ Plasmids expressing multiple gRNAs were designed and constructed using a recently published method.^14^

### Multiplexed Expression of gRNAs Increases *trp1* Reversion Rates

We introduced a premature stop codon into the *TRP1* gene (GAG → tAG, *trp1* E23*), a gene involved in biosynthesis of tryptophan, whose inactivation renders cells auxotrophic for tryptophan. The frequency of reversion to tryptophan prototrophy (Trp^+^) served as a readout of mutagenesis by yEvolvR. The trp1 E23* locus was targeted by four gRNAs which flank the premature stop codon (Figure 1B). All gRNAs bind within a ∼100 bp long stretch of DNA, defining the targeted window. The gRNAs have a comparable GC content (45–55%), and comparable on-target and off-target scores when evaluated using a previously published model^15^ (Supplementary Table S5). We evaluated the on-target *trp1* reversion frequency using individual gRNAs (*TRP1* gRNA1, gRNA2, gRNA3, gRNA4), two combinations of two gRNAs (*TRP1* gRNA1 + 4 and *gRNA2 + 3*), and a combination of all four gRNAs (*TRP1* gRNA1 + 2 + 3 + 4) for both the yEvolvR(D10A) and the yEvolvR(H840A) variant. A gRNA targeting *CAN1* (*CAN1* gRNA7) served as an off-target control (Figure 1C). The functionality of individual gRNAs was confirmed by performing *TRP1* and *CAN1* knockouts, respectively, using Cas9 (data not shown). For the *trp1* reversion assay, yeast was transformed with plasmids encoding *TEF1p*-yEvolvR, *csy4*, and gRNA(s). After 72 h of growth, the amount of Trp^+^ cells was quantified, and the fraction of Trp^+^ cells per total cells was calculated (*trp1* reversion frequency). In addition to reversion, Trp^+^ cells can also arise when the stop codon is suppressed by tRNA mutations, similar to the *SUP4-o* mutant.^16^ We cannot distinguish between these two effects (reversion and suppression) with this assay; however, we assume that suppression frequencies are constant and independent of the used gRNA, enabling a comparison between mutation frequencies with different gRNAs.

Individually expressed, gRNAs *TRP1* gRNA1 and *TRP1* gRNA3 led to low reversion frequencies, while *TRP1* gRNA2 and *TRP1* gRNA4 yielded high reversion frequencies. Expression of the off-target gRNA *CAN1* gRNA7 resulted in the lowest observed *trp1* reversion frequency. Reversion frequencies for the pairs of gRNAs, *TRP1* gRNA1 + 4 and TRP1 gRNA2 + 3, were comparable to the reversion frequencies of the individually expressed gRNAs *TRP1* gRNA4 and *TRP1* gRNA2, respectively. When expressing all four gRNAs simultaneously (*TRP1* gRNA1 + 2 + 3 + 4), a further increase in reversion frequency was observed for yEvolvR(H840A), but not for yEvolvR(D10A). We also evaluated on-target and off-target reversion frequencies in a strain with a partially defective DNA mismatch repair MMR, generated by deletion of *MSH6* (Figure 1D). While the off-target reversion frequency was increased 1 order of magnitude in the *msh6Δ* background, we observed no significant increase for the on-target reversion frequency when expressing all four gRNAs simultaneously (*TRP1* gRNA1 + 2 + 3 + 4).

To enable temporal control of mutagenesis, we constructed an inducible yEvolvR variant. A copy of yEvolvR(H840A) under control of the *GAL1p* promoter was genomically integrated into integration site X-4.^17^ The setup for expression of *csy4* and gRNAs remained as described previously. A time-course experiment of *trp1* reversion was performed (Figure 1E). No increase in reversion frequency was observed when cells were incubated for 72 h on glucose under noninducing conditions. When cells were incubated on galactose under inducing conditions, cells expressing the gRNAs *TRP1* gRNA1 + 2 + 3 + 4 showed an increased *trp1* reversion frequency after 24 h, which plateaued after 48 h. No increase was observed for cells expressing gRNA7 targeting *CAN1.* Compared to results obtained from the centromeric *TEF1p*-EvolvR plasmid, the observed on-target mutation frequency was slightly lower, while the observed off-target frequency was comparable.

### Amplicon Sequencing Reveals a Synergistic Effect of Multiple gRNAs on Mutagenesis

**Figure 2:**
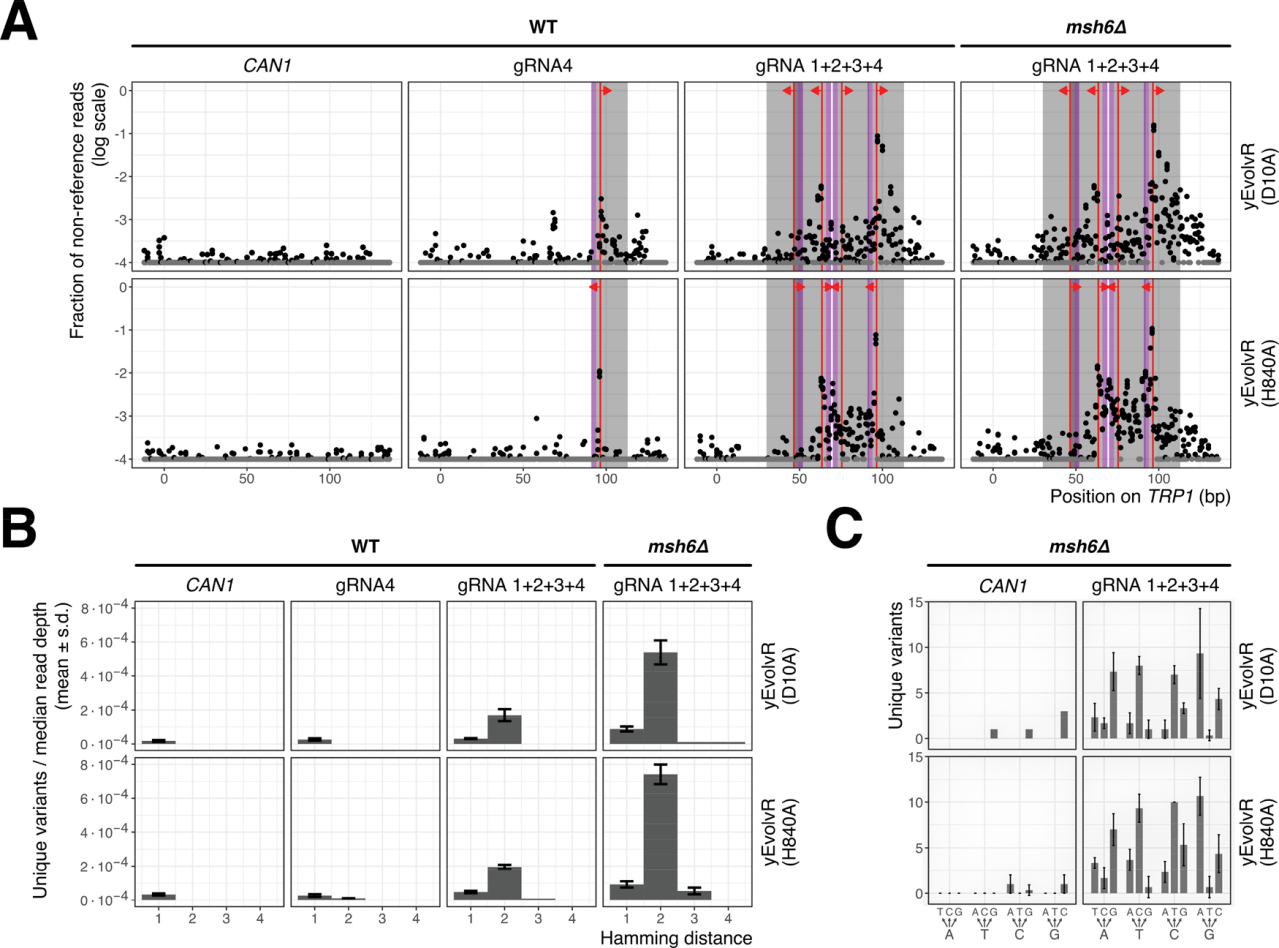
Synergistic effect of expression of multiple gRNAs on mutagenesis with yEvolvR. (A) Mutation frequency as a function of position in the gene. Yeast cells were transformed with plasmids encoding yEvolvR, *csy4*, and gRNA(s). Liquid minimal medium containing tryptophan was directly inoculated with the transformants. After 72 h of growth, cells were harvested, genomic DNA was isolated, and the *trp1* locus was amplified. Untransformed cells served as a control. Gray box: Target sequence, purple box: protospacer adjacent motif, red line: nick site, red arrow: direction of yEvolvR activity. (B) Hamming distance of unique reads (number of occurrence ≥5 reads) normalized by mean read depth. (C) Unique variants (number of occurrence ≥5 reads) found in indicated samples. Samples had an average read depth of 96322.

Since the phenotypic assay for *trp1* reversion selects only for functional mutations that result in tryptophan prototrophy, we performed amplicon sequencing of the targeted window within *trp1* E23* to get a complete picture of the occurring mutations. Yeast cells were transformed with plasmids encoding *TEF1p*-yEvolvR, *csy4*, and gRNA(s). Liquid minimal medium containing tryptophan was directly inoculated with the transformants. After 72 h of growth, cells were harvested, genomic DNA was isolated, and the *trp1* E23* locus was amplified. Untransformed cells served as an additional control. Compared to the off-target gRNA control, an elevated frequency of mutagenesis around the target DNA sequence was observed for on-target gRNAs (Figure 2A and Supplementary Figure S1). This increase was most obvious for gRNAs that resulted in high *trp1* reversion frequencies. As expected, mutation frequencies were highest for bases close to the nick introduced by the nickase. The first base in direction of yEvolvR activity adjacent to the nick is most likely to be mutated by PolI5M.^8^ The frequency of mutagenesis was strongly increased in samples with multiplexed gRNA expression of all four gRNAs, both for yEvolvR(D10A) and yEvolvR(H840A), with up to 18% of the reads containing mutations. The mutation frequency in samples with multiplexed gRNA expression of all four gRNAs was greater than the sum of mutation frequencies in samples with individual gRNA expression, pointing toward a synergistic effect (Figure 2, Supplementary Figure S1).

This effect was even more pronounced in the *msh6Δ* background, with up to 27% reads containing mutations. The direction of yEvolvR activity was clearly visible in these samples, with mutations occurring primarily 3′ of the introduced nick. However, increased mutation frequencies in the opposite direction were also observed, consistent with earlier reports.^5^ We removed indels from our data set and calculated the Hamming distance of remaining unique reads with an occurrence of ≥5 reads in our samples, normalized by median read depth, to describe the generated genetic diversity (Figure 2B). The Hamming distance is a metric for similarity between pairs of sequences. The distance describes the minimum number of base substitutions required to transform a mutated sequence to the reference sequence. We observed an increased abundance of unique reads with a Hamming distance of >1 in multiplexed samples. The average Hamming distance is increased in multiplexed samples. This is more clearly observed in a *msh6Δ* background. In multiplexed samples of yEvolvR(H840A), the average distance is further increased. We found that all four nucleotides were mutated, and we observed all possible 12 transitions and transversions (Figure 2C).

### Multiplex Expression of gRNAs and Induction of Mutagenesis Improve the Capabilities of yEvolvR

Building on previous work with yEvolvR in *S. cerevisiae* as eukaryotic cellular host, we have shown that multiplexed expression of gRNAs can improve mutation frequencies in a targeted window. We have demonstrated that deletion of the MMR component *MSH6* further improves mutation frequencies. Finally, we have characterized an inducible yEvolvR system, which enables temporal control of mutagenesis.

The efficiency of mutagenesis when expressing individual gRNAs is highly dependent on the gRNA. While gRNAs *TRP1* gRNA1 and *TRP1* gRNA3 yielded low reversion frequencies, *TRP1* gRNA2 and *TRP1* gRNA4 yielded high reversion frequencies. The same trend was observed when analyzing mutation frequencies by amplicon sequencing. The criteria that determine efficiency are unclear, as we observe no clear correlation between mutation frequencies and the gRNAs’ distance to the premature stop codon, GC content, strand localization, on-target score, Gibbs free energy of guide sequence secondary structure formation, or capability for extended stem-loop formation.^15,18−20^ The efficiency of mutagenesis might be more dependent on the DNA sequence around the gRNA binding site, as has been observed for the repair profiles of Cas9-generated indels.^21^ Predictions for the repair outcomes of Cas9-induced double-strand breaks are improving,^22^ and similar efforts are required to accurately predict mutation outcomes for yEvolvR with different gRNAs.

Multiplexed expression of gRNAs increased the rate of mutagenesis. This synergistic effect is likely due to changes in the involved DNA repair mechanisms. *TRP1* gRNA2 and *TRP1* gRNA4 are localized on opposite strands. While expression of individual gRNAs results in nicking of a single DNA strand, expression of both gRNAs simultaneously results in nicking of both DNA strands, creating a DNA double-strand break. The repair of DNA single-strand breaks and DNA double-strand breaks involves different DNA repair mechanisms with different fidelities. A similar double nicking strategy has been described previously for improving Cas9 editing specificity in mammalian cells.^23,24^ While multiplexing increased mutation frequencies for both yEvolvR variants, the increase was more pronounced for the H840A variant. For this variant, when expressing *TRP1* gRNA2 and *TRP1* gRNA4, the directions of yEvolvR activity are directed toward each other, which might result in a synergistic effect on mutation rates. This notion is supported by the increased average Hamming distance in these samples. Increasing the number of expressed gRNAs could further increase efficiency. While this study multiplexed up to 4 gRNAs, multiplexing of up to 12 gRNAs has been reported.^25^

While deletion of *MSH6* greatly increased on-target mutation frequency, it also increased off-target mutation frequency by more than 1 order of magnitude. This undesired increase might be circumvented by downregulation, rather than deletion, of *MSH6*. Mismatch recognition can also be disrupted by overexpression of the dominant-negative mutant *msh6* F337A.^26^ Similarly, the MMR component *MLH1* has been inactivated by overexpression of a dominant-negative mutant in mammalian cells, and by overexpression of the wild-type in yeast.^27,28^ We demonstrated that yEvolvR under control of the *GAL1p* promoter enables temporal control of mutagenesis by induction of expression through addition of galactose. The observed off-target frequency for *GAL1p*-controlled inducible expression is comparable to constitutive, *TEF1p*-controlled expression of yEvolvR.

While multiplexing of gRNA expression and the associated increase in mutation frequencies represents an improvement of yEvolvR, there are remaining challenges. In the current model for yEvolvR activity, the Cas9 nickase must dissociate from the target DNA sequence after DNA cleavage before DNA Polymerase I can bind and perform strand displacement synthesis. However, Cas9-gRNA complexes from *S. pyogenes* remain tightly bound to the target DNA sequence after DNA cleavage, effectively preventing mutagenesis of the target sequence by PolI5M.^30,31^ Interestingly, Cas9-gRNA complexes from *Staphylococcus aureus* partially release the target DNA sequence after DNA cleave, abolishing the strong interaction.^32^ A nickase derived from this Cas9 variant dissociates faster from the target DNA sequence, which could enable more frequent binding of the DNA Polymerase I, and thereby potentially improving mutagenesis frequencies when using yEvolvR. After selecting a targeted window for mutation (e.g., a regulatory site, substrate binding site), multiplexed expression of gRNAs and yEvolvR can create a diverse library *in vivo*. gRNAs should be chosen with the yEvolvR variant and the associated direction of yEvolvR activity in mind.

While other *in vivo* mutagenesis methods appear more practical for diversification of longer stretches of DNA, the improved method presented here is particularly suited for generating high mutation frequencies in a short stretch of DNA. Our multiplexed yEvolvR approach achieves mutation frequencies of up to 5 × 10^–2^ mutations per bp.

## Methods

### Plasmid and Strain Construction

Plasmids were constructed via Golden Gate assembly (using parts created in this study and from the MoClo collection), by Gibson assembly, or by site-directed mutagenesis of previously constructed plasmids.^17,33,34^*E. coli* and *S. cerevisiae* cells were transformed using standard molecular biology techniques. Genome modifications of yeast were performed in a Cas9-dependent manner. The strain YEY1 was generated by introducing a premature stop codon into *TRP1*, generating trp1 E23*. YEY2 was generated by deleting *MSH6* in YEY1. Due to toxicity issues when attempting to construct a plasmid encoding *GAL1p*-yEvolvR(H840A) in *E. coli*, the corresponding Golden Gate assembly was used as a template to amplify *GAL1p*-yEvolvR(H840A) with overlap for integration site X-4^35^ and was then integrated into YEY1, generating YEY3. Plasmids are listed in Supplementary Table S1, strains are listed in Supplementary Table S2, oligonucleotides are listed in Supplementary Table S3, and dsDNA fragments are listed in Supplementary Table S4. After reevaluation of Sanger sequencing data for the plasmid encoding pTEF1-yEvolvR(H840A) (pMG356), we noticed that during construction of the yeast expression plasmid, we had picked up an additional mutation, G752S, in the enCas9 part. To ensure that this additional mutation did not affect the function of enCas9, the H840A mutant was reconstructed and was shown to behave comparably to the G752S H840A mutant in a *trp1 reversion* assay (Supplementary Figure S2).

### *trp1* Reversion Assay

For determination of *trp1* reversion frequency using constitutive expression of yEvolvR, YEY1 and YEY2 were transformed with 500 ng of either pMG355 or pMG356, together with 500 ng of gRNA plasmid. Cells were resuspended in 50 μL sterile water, and 10 μL of cells were used to inoculate 2.5 mL synthetic medium containing 2% glucose, in triplicate, in a 24-deep well plate. The synthetic medium consisted of 7.5 g/L (NH4)_2_SO_4_, 14.4 g/L KH_2_PO_4_, 0.5 g/L MgSO_4_·7H_2_O, 1 mL/L vitamin mix, 2 mL/L trace metal solution, pH adjusted to 6.5. Trace metal solution contained 3.0 g/L FeSO_4_·7H_2_O, 4.5 g/L ZnSO_4_·7H_2_O, 4.5 g/L CaCl_2_·2H_2_O, 1.0 g/L MnCl_2_·4H_2_O, 0.3 g/L CoCl_2_·6H_2_O, 0.3 g/L CuSO_4_·5H_2_O, 0.4 g/L Na_2_MoO_4_·2H_2_O, 1.0 g/L H_3_BO_3_, 0.1 g/L KI, and 19.0 g/L EDTA disodium salt dihydrate. Vitamin solution contained 0.05 g/L biotin, 1.0 g/L d-pantothenic acid hemicalcium salt, 1.0 g/L thiamine-HCl, 1.0 g/L pyridoxine-HCl, 1.0 g/L nicotinic acid, 0.2 g/L 4-aminobenzoic acid, and 25 g/L myo-inositol. The medium was supplied with 75 mg/L l-tryptophan. Cells were then cultivated for 72 h before plating cells on SD-Ura-His and SD-Trp agar plates to determine colony forming units (CFU). The *trp1* reversion frequency was calculated as Trp^+^ CFUs divided by total CFUs.

For determination of *trp1* reversion frequency using inducible expression of yEvolvR, YEY3 was transformed with 100 ng of *TRP1* gRNA1 + 2 + 3 + 4 and 100 ng of pMG382. Transformants were first incubated overnight in synthetic medium containing 2% glucose, and then overnight in synthetic medium containing 2% raffinose. The raffinose preculture was then used to inoculate 2.5 mL synthetic medium containing 2% galactose to a starting OD600 = 0.1, in triplicate, in a 24-deep well plate. All medium was supplied with 75 mg/L l-tryptophan.^36^ Cells were then cultivated for 72 h and the *trp1* reversion frequency was calculated as Trp^+^ CFUs divided by total CFUs.

### Next Generation Sequencing (NGS)

Cells were cultivated as described before. Two milliliters of cells were harvested after 72 h, washed once with water, and the cell pellets were stored at −20 °C. Additionally, two mL of cells from the preculture used for transformation were harvested, which served as an additional control. Genomic DNA was isolated using a LiOAc method (Lõoke *et al.*, 2011).^37a^ DNA concentration was determined using Qubit and the Qubit dsDNA HS assay kit. 40 ng of genomic DNA served as template for the first PCR (20 cycles). The second PCR was performed using a Nextera Indexing Kit V2 Set A (15 cycles). The PCRs were purified using Ampure XP beads, diluted to 10 nM, pooled, and quality was controlled by analyzing the pooled library on a Bioanalyzer High Sensitivity DNA analysis kit. MiSeq 2 × 150 bp sequencing was performed at SciLifeLab, Stockholm.

### NGS Data Analysis

The sequenced region on the *TRP1* locus was short enough to allow complete overlap of complementary paired-end reads. Using NGmerge,^37^ paired-end reads in FASTQ format with perfectly overlapping complementary regions were merged using the following parameters: mismatches to allow in the overlapping region = 0, FASTQ quality offset = 33, and maximum input quality score = 40. Merged reads were subsequently aligned to the *TRP1* locus using the burrows-wheeler aligner^38^ and the resulting alignment files in BAM format were then sorted using SAMtools sort.^39^ Next, sorted BAM files were piled up into the tabular VCF format, containing counts of all detected genetic variants across samples, using BCFtools mpileup3 with the parameter maximum depth = 600000. The entire pipeline for producing VCF files from raw FASTQ files was implemented using the Snakemake workflow engine^40^ and package versions were managed using Conda.

Processing of the resulting VCF file was performed in the R software package (version 4.1.2) along with the Tidyverse suite of packages.^41^ The resulting VCF file was parsed into a tidy format containing allele counts for each position on the *TRP1* gene across all samples. Regions containing a read depth of less than 40000 were discarded from the analysis. This resulted in one sample (replicate #3 of the strain containing the CAN1 targeting gRNA in the msh6Δ background) being removed from subsequent analyses. End regions of the *TRP1* gene were also removed due to reduced quality of base calling from NGS. Counts of nonreference bases at each position were then normalized for sequencing depth in order to calculate the proportion of mutated bases at each position across samples. These values were then subtracted by the proportion of mutated bases in the parental WT samples in order to highlight regions enriched for mutations, values with a proportion of alternative bases less than 10^–4^ were set to zero. The depth normalized and background subtracted values for each position were then plotted along the positions on *TRP1*. The fraction of nonreference reads is defined as the mutation frequency.

In order to gain read-level information, e.g., number and type of SNPs within individual reads, sample BAM files obtained from the alignment step were merged into one file using SAMtools merge. The merged BAM file was then trimmed in order to obtain 160 bp from the left, and 170 bp from the right using trimBam from the BamUtil suite (https://github.com/statgen/bamUtil). The trimmed bam file was subsequently converted into FASTA format and tab characters in the sample names introduced by trimBam were removed using the following sed command: sed -i.bak -E $’s/\t//all_trimmed_noTab.fasta. This processed FASTA file was then split into 100 smaller files (in order to conserve RAM) using splitfasta (https://pypi.org/project/split-fasta/), with all files being subsequently imported into R one-by-one using the Biostrings package.^42^ Only unique reads were tallied up kept for subsequent analyses. Hamming/edit distances between the reference and the unique reads were calculated and used to remove reads containing indels based on mismatches between each reads hamming and edit distance. The number of unique reads in each sample were then normalized their corresponding read-depth and reads with occurrences ≥5 in the parental WT strains were removed from daughter strains. Normalized unique reads were then plotted for all samples against the reads corresponding hamming distance to the reference sequence. The number of SNPs obtained from the unique reads were grouped by transition/transversion type and plotted for all samples. All computational analyses were performed on a 2020 MacBook Pro with 4 cores [Intel Core 10th generation CPU @4.1 GHz] and 16 GB RAM running MacOS Monterey version 12.5.1.

## Data Availability

All code and data used in the analysis is available at https://github.com/angelolimeta/BE-VCF.

## Supporting Information Available

Supplementary figures that include detailed results for amplicon sequencing for individual gRNAs (Figure S1) and *trp1* reversion frequencies for yEvolvR(G752S H840A) (PDF)
Supplementary tables that include information about plasmids (Table S1), strains (Table S2), oligonucleotides (Table S3), dsDNA fragments (Table S4), and gRNA characteristics (Table S5) used in this study (XLSX)

## References

[ref1] Cravens A.; Jamil O. K.; Kong D.; Sockolosky J. T.; Smolke C. D. Polymerase-Guided Base Editing Enables in Vivo Mutagenesis and Rapid Protein Engineering. Nat. Commun. 2021, 12 (1), 1579 10.1038/s41467-021-21876-z. 33707425 PMC7952560

[ref2] Crook N.; Abatemarco J.; Sun J.; Wagner J. M.; Schmitz A.; Alper H. S. In Vivo Continuous Evolution of Genes and Pathways in Yeast. Nat. Commun. 2016, 7 (1), 13051 10.1038/ncomms13051. 27748457 PMC5071640

[ref3] Nishida K.; Arazoe T.; Yachie N.; Banno S.; Kakimoto M.; Tabata M.; Mochizuki M.; Miyabe A.; Araki M.; Hara K. Y.; Shimatani Z.; Kondo A. Targeted Nucleotide Editing Using Hybrid Prokaryotic and Vertebrate Adaptive Immune Systems. Science 2016, 353 (6305), aaf8729 10.1126/science.aaf8729. 27492474

[ref4] Ravikumar A.; Arzumanyan G. A.; Obadi M. K. A.; Javanpour A. A.; Liu C. C. Scalable, Continuous Evolution of Genes at Mutation Rates above Genomic Error Thresholds. Cell 2018, 175 (7), 1946–1957. 10.1016/j.cell.2018.10.021. 30415839 PMC6343851

[ref5] Tou C. J.; Schaffer D. V.; Dueber J. E. Targeted Diversification in the *S. Cerevisiae* Genome with CRISPR-Guided DNA Polymerase I. ACS Synth. Biol. 2020, 9 (7), 1911–1916. 10.1021/acssynbio.0c00149. 32485105

[ref6] Jinek M.; Chylinski K.; Fonfara I.; Hauer M.; Doudna J. A.; Charpentier E. A Programmable Dual-RNA–Guided DNA Endonuclease in Adaptive Bacterial Immunity. Science 2012, 337 (6096), 816–821. 10.1126/science.1225829. 22745249 PMC6286148

[ref7] García-García J. D.; Joshi J.; Patterson J. A.; Trujillo-Rodriguez L.; Reisch C. R.; Javanpour A. A.; Liu C. C.; Hanson A. D. Potential for Applying Continuous Directed Evolution to Plant Enzymes: An Exploratory Study. Life 2020, 10 (9), 179 10.3390/life10090179. 32899502 PMC7555113

[ref8] Halperin S. O.; Tou C. J.; Wong E. B.; Modavi C.; Schaffer D. V.; Dueber J. E. CRISPR-Guided DNA Polymerases Enable Diversification of All Nucleotides in a Tunable Window. Nature 2018, 560 (7717), 248–252. 10.1038/s41586-018-0384-8. 30069054

[ref9] Slaymaker I. M.; Gao L.; Zetsche B.; Scott D. A.; Yan W. X.; Zhang F. Rationally Engineered Cas9 Nucleases with Improved Specificity. Science 2016, 351 (6268), 84–88. 10.1126/science.aad5227. 26628643 PMC4714946

[ref10] Johnson R. E.; Kovvali G. K.; Prakash L.; Prakash S. Requirement of the Yeast MSH3 and MSH6 Genes for MSH2-Dependent Genomic Stability. J. Biol. Chem. 1996, 271 (13), 7285–7288. 10.1074/jbc.271.13.7285. 8631743

[ref11] Marsischky G. T.; Filosi N.; Kane M. F.; Kolodner R. Redundancy of Saccharomyces Cerevisiae MSH3 and MSH6 in MSH2-Dependent Mismatch Repair. Genes Dev. 1996, 10 (4), 407–420. 10.1101/gad.10.4.407. 8600025

[ref12] Ferreira R.; Skrekas C.; Nielsen J.; David F. Multiplexed CRISPR/Cas9 Genome Editing and Gene Regulation Using Csy4 in. Saccharomyces Cerevisiae. ACS Synth. Biol. 2018, 7 (1), 10–15. 10.1021/acssynbio.7b00259. 29161506

[ref13] Lian J.; Bao Z.; Hu S.; Zhao H. Engineered CRISPR/Cas9 System for Multiplex Genome Engineering of Polyploid Industrial Yeast Strains. Biotechnol. Bioeng. 2018, 115 (6), 1630–1635. 10.1002/bit.26569. 29460422

[ref14] Otto M.; Skrekas C.; Gossing M.; Gustafsson J.; Siewers V.; David F. Expansion of the Yeast Modular Cloning Toolkit for CRISPR-Based Applications, Genomic Integrations and Combinatorial Libraries. ACS Synth. Biol. 2021, 10 (12), 3461–3474. 10.1021/acssynbio.1c00408. 34860007 PMC8689691

[ref15] Doench J. G.; Fusi N.; Sullender M.; Hegde M.; Vaimberg E. W.; Donovan K. F.; Smith I.; Tothova Z.; Wilen C.; Orchard R.; Virgin H. W.; Listgarten J.; Root D. E. Optimized SgRNA Design to Maximize Activity and Minimize Off-Target Effects of CRISPR-Cas9. Nat. Biotechnol. 2016, 34 (2), 184–191. 10.1038/nbt.3437. 26780180 PMC4744125

[ref16] Goodman H. M.; Olson M. V.; Hall B. D. Nucleotide Sequence of a Mutant Eukaryotic Gene: The Yeast Tyrosine-Inserting Ochre Suppressor SUP4-o. Proc. Natl. Acad. Sci. U. S. A. 1977, 74 (12), 5453–5457. 10.1073/pnas.74.12.5453. 341157 PMC431761

[ref17] Jessop-Fabre M. M.; Jakočiu̅nas T.; Stovicek V.; Dai Z.; Jensen M. K.; Keasling J. D.; Borodina I. EasyClone-MarkerFree: A Vector Toolkit for Marker-less Integration of Genes into *Saccharomyces Cerevisiae* via CRISPR-Cas9. Biotechnol. J. 2016, 11 (8), 1110–1117. 10.1002/biot.201600147. 27166612 PMC5094547

[ref18] Clarke R.; Heler R.; MacDougall M. S.; Yeo N. C.; Chavez A.; Regan M.; Hanakahi L.; Church G. M.; Marraffini L. A.; Merrill B. J. Enhanced Bacterial Immunity and Mammalian Genome Editing via RNA-Polymerase-Mediated Dislodging of Cas9 from Double-Strand DNA Breaks. Mol. Cell 2018, 71 (1), 42–55. 10.1016/j.molcel.2018.06.005. 29979968 PMC6063522

[ref19] Jensen K. T.; Fløe L.; Petersen T. S.; Huang J.; Xu F.; Bolund L.; Luo Y.; Lin L. Chromatin Accessibility and Guide Sequence Secondary Structure Affect CRISPR-Cas9 Gene Editing Efficiency. FEBS Lett. 2017, 591 (13), 1892–1901. 10.1002/1873-3468.12707. 28580607

[ref20] Wong N.; Liu W.; Wang X. WU-CRISPR: Characteristics of Functional Guide RNAs for the CRISPR/Cas9 System. Genome Biol. 2015, 16 (1), 218 10.1186/s13059-015-0784-0. 26521937 PMC4629399

[ref21] Lemos B. R.; Kaplan A. C.; Bae J. E.; Ferrazzoli A. E.; Kuo J.; Anand R. P.; Waterman D. P.; Haber J. E. CRISPR/Cas9 Cleavages in Budding Yeast Reveal Templated Insertions and Strand-Specific Insertion/Deletion Profiles. Proc. Natl. Acad. Sci. U. S. A. 2018, 115 (9), E2040–E2047. 10.1073/pnas.1716855115. 29440496 PMC5834694

[ref22] Allen F.; Crepaldi L.; Alsinet C.; Strong A. J.; Kleshchevnikov V.; De Angeli P.; Páleníková P.; Khodak A.; Kiselev V.; Kosicki M.; Bassett A. R.; Harding H.; Galanty Y.; Muñoz-Martínez F.; Metzakopian E.; Jackson S. P.; Parts L. Predicting the Mutations Generated by Repair of Cas9-Induced Double-Strand Breaks. Nat. Biotechnol. 2019, 37 (1), 64–72. 10.1038/nbt.4317. PMC6949135 30480667

[ref23] Mali P.; Aach J.; Stranges P. B.; Esvelt K. M.; Moosburner M.; Kosuri S.; Yang L.; Church G. M. CAS9 Transcriptional Activators for Target Specificity Screening and Paired Nickases for Cooperative Genome Engineering. Nat. Biotechnol. 2013, 31 (9), 833–838. 10.1038/nbt.2675. 23907171 PMC3818127

[ref24] Ran F. A.; Hsu P. D.; Lin C.-Y.; Gootenberg J. S.; Konermann S.; Trevino A. E.; Scott D. A.; Inoue A.; Matoba S.; Zhang Y.; Zhang F. Double Nicking by RNA-Guided CRISPR Cas9 for Enhanced Genome Editing Specificity. Cell 2013, 154 (6), 1380–1389. 10.1016/j.cell.2013.08.021. 23992846 PMC3856256

[ref25] McCarty N. S.; Shaw W. M.; Ellis T.; Ledesma-Amaro R. Rapid Assembly of GRNA Arrays *via* Modular Cloning in Yeast. ACS Synth. Biol. 2019, 8 (4), 906–910. 10.1021/acssynbio.9b00041. 30939239

[ref26] Bowers J.; Sokolsky T.; Quach T.; Alani E. A Mutation in the MSH6 Subunit of the Saccharomyces Cerevisiae MSH2-MSH6 Complex Disrupts Mismatch Recognition. J. Biol. Chem. 1999, 274 (23), 16115–16125. 10.1074/jbc.274.23.16115. 10347163

[ref27] Chen P. J.; Hussmann J. A.; Yan J.; Knipping F.; Ravisankar P.; Chen P.-F.; Chen C.; Nelson J. W.; Newby G. A.; Sahin M.; Osborn M. J.; Weissman J. S.; Adamson B.; Liu D. R. Enhanced Prime Editing Systems by Manipulating Cellular Determinants of Editing Outcomes. Cell 2021, 184 (22), 5635–5652. 10.1016/j.cell.2021.09.018. 34653350 PMC8584034

[ref28] Shcherbakova P. V.; Hall M. C.; Lewis M. S.; Bennett S. E.; Martin K. J.; Bushel P. R.; Afshari C. A.; Kunkel T. A. Inactivation of DNA Mismatch Repair by Increased Expression of Yeast *MLH1*. Mol. Cell. Biol. 2001, 21 (3), 940–951. 10.1128/MCB.21.3.940-951.2001. 11154280 PMC86684

[ref30] Raper A. T.; Stephenson A. A.; Suo Z. Functional Insights Revealed by the Kinetic Mechanism of CRISPR/Cas9. J. Am. Chem. Soc. 2018, 140 (8), 2971–2984. 10.1021/jacs.7b13047. 29442507

[ref31] Sternberg S. H.; Redding S.; Jinek M.; Greene E. C.; Doudna J. A. DNA Interrogation by the CRISPR RNA-Guided Endonuclease Cas9. Nature 2014, 507 (7490), 62–67. 10.1038/nature13011. 24476820 PMC4106473

[ref32] Zhang S.; Zhang Q.; Hou X.; Guo L.; Wang F.; Bi L.; Zhang X.; Li H.; Wen F.; Xi X.; Huang X.; Shen B.; Sun B. Dynamics of *Staphylococcus Aureus* Cas9 in DNA Target Association and Dissociation. EMBO Rep. 2020, 10.15252/embr.202050184. PMC7534634 32790142

[ref33] Lee M. E.; DeLoache W. C.; Cervantes B.; Dueber J. E. A Highly Characterized Yeast Toolkit for Modular, Multipart Assembly. ACS Synth. Biol. 2015, 4 (9), 975–986. 10.1021/sb500366v. 25871405

[ref34] Hellgren J.; Godina A.; Nielsen J.; Siewers V. Promiscuous Phosphoketolase and Metabolic Rewiring Enables Novel Non-Oxidative Glycolysis in Yeast for High-Yield Production of Acetyl-CoA Derived Products. Metab. Eng. 2020, 62, 150–160. 10.1016/j.ymben.2020.09.003. 32911054

[ref35] Mikkelsen M. D.; Buron L. D.; Salomonsen B.; Olsen C. E.; Hansen B. G.; Mortensen U. H.; Halkier B. A. Microbial Production of Indolylglucosinolate through Engineering of a Multi-Gene Pathway in a Versatile Yeast Expression Platform. Metab. Eng. 2012, 14 (2), 104–111. 10.1016/j.ymben.2012.01.006. 22326477

[ref36] Pronk J. T. Auxotrophic Yeast Strains in Fundamental and Applied Research. Appl. Environ. Microbiol. 2002, 68 (5), 2095–2100. 10.1128/AEM.68.5.2095-2100.2002. 11976076 PMC127579

[ref37a] Lõoke M.; Kristjuhan K.; Kristjuhan A. Extraction of genomic DNA from yeasts for PCR-based applications. Biotechniques 2011, 50 (5), 325–328. 10.2144/000113672. 21548894 PMC3182553

[ref37] Gaspar J. M. NGmerge: Merging Paired-End Reads via Novel Empirically-Derived Models of Sequencing Errors. BMC Bioinformatics 2018, 19 (1), 536 10.1186/s12859-018-2579-2. 30572828 PMC6302405

[ref38] Li H.; Durbin R. Fast and Accurate Short Read Alignment with Burrows-Wheeler Transform. Bioinformatics 2009, 25 (14), 1754–1760. 10.1093/bioinformatics/btp324. 19451168 PMC2705234

[ref39] Danecek P.; Bonfield J. K.; Liddle J.; Marshall J.; Ohan V.; Pollard M. O.; Whitwham A.; Keane T.; McCarthy S. A.; Davies R. M.; Li H. Twelve Years of SAMtools and BCFtools. GigaScience 2021, 10 (2), giab008 10.1093/gigascience/giab008. 33590861 PMC7931819

[ref40] Mölder F.; Jablonski K. P.; Letcher B.; Hall M. B.; Tomkins-Tinch C. H.; Sochat V.; Forster J.; Lee S.; Twardziok S. O.; Kanitz A.; Wilm A.; Holtgrewe M.; Rahmann S.; Nahnsen S.; Köster J. Sustainable Data Analysis with Snakemake. F1000Research 2021, 10, 33 10.12688/f1000research.29032.2. 34035898 PMC8114187

[ref41] Wickham H.; Averick M.; Bryan J.; Chang W.; McGowan L.; François R.; Grolemund G.; Hayes A.; Henry L.; Hester J.; Kuhn M.; Pedersen T.; Miller E.; Bache S.; Müller K.; Ooms J.; Robinson D.; Seidel D.; Spinu V.; Takahashi K.; Vaughan D.; Wilke C.; Woo K.; Yutani H. Welcome to the Tidyverse. J. Open Source Softw. 2019, 4 (43), 1686 10.21105/joss.01686.

[ref42] Pagès H.; Aboyoun P.; Gentleman R.; DebRoy S. Biostrings: Efficient Manipulation of Biological Strings. https://bioconductor.org/packages/Biostrings.

