# Supplementary material for: Multiplexed Guide RNA Expression Leads to Increased Mutation Frequency in Targeted Window Using a CRISPR-Guided Error-Prone DNA Polymerase in Saccharomyces cerevisiae
Source: ACS Synth Biol. 2023 Jul 24;12(8):2271–7. doi: 10.1021/acssynbio.2c00689 (PMC10443033; doi:10.1021/acssynbio.2c00689)
Supplement: Supplementary file 1 — sb2c00689_si_001.pdf [file sb2c00689_si_001.pdf]

Support information

Multiplexed guide RNA expression leads to increased mutation frequency in targeted window using a CRISPR-guided error-prone DNA polymerase in *Saccharomyces cerevisiae*

Michael Gossing[1,] Angelo Limeta[2], Christos Skrekas[2], Mark Wigglesworth[4,6], Andrew Davis[5], Verena Siewers[2,3], Florian David*[2]

[1]Discovery Sciences, Biopharmaceuticals R&D, AstraZeneca, SE-41320 Gothenburg, Sweden

[2]Department of Life Sciences, Chalmers University of Technology, SE-41296 Gothenburg, Sweden

[3]Novo Nordisk Foundation Center for Biosustainability, Technical University of Denmark, DK-2800 Kgs. Lyngby, Denmark

[4]Discovery Sciences, Biopharmaceuticals R&D, AstraZeneca, Alderley Park SK10 2NA, U.K.

[5]Discovery Sciences, Biopharmaceutical R&D, AstraZeneca, Cambridge, CB2 0AA, U.K.

[6]Alderley Lighthouse Labs Ltd, Alderley Park, SK10 4TG Macclesfield, U.K.

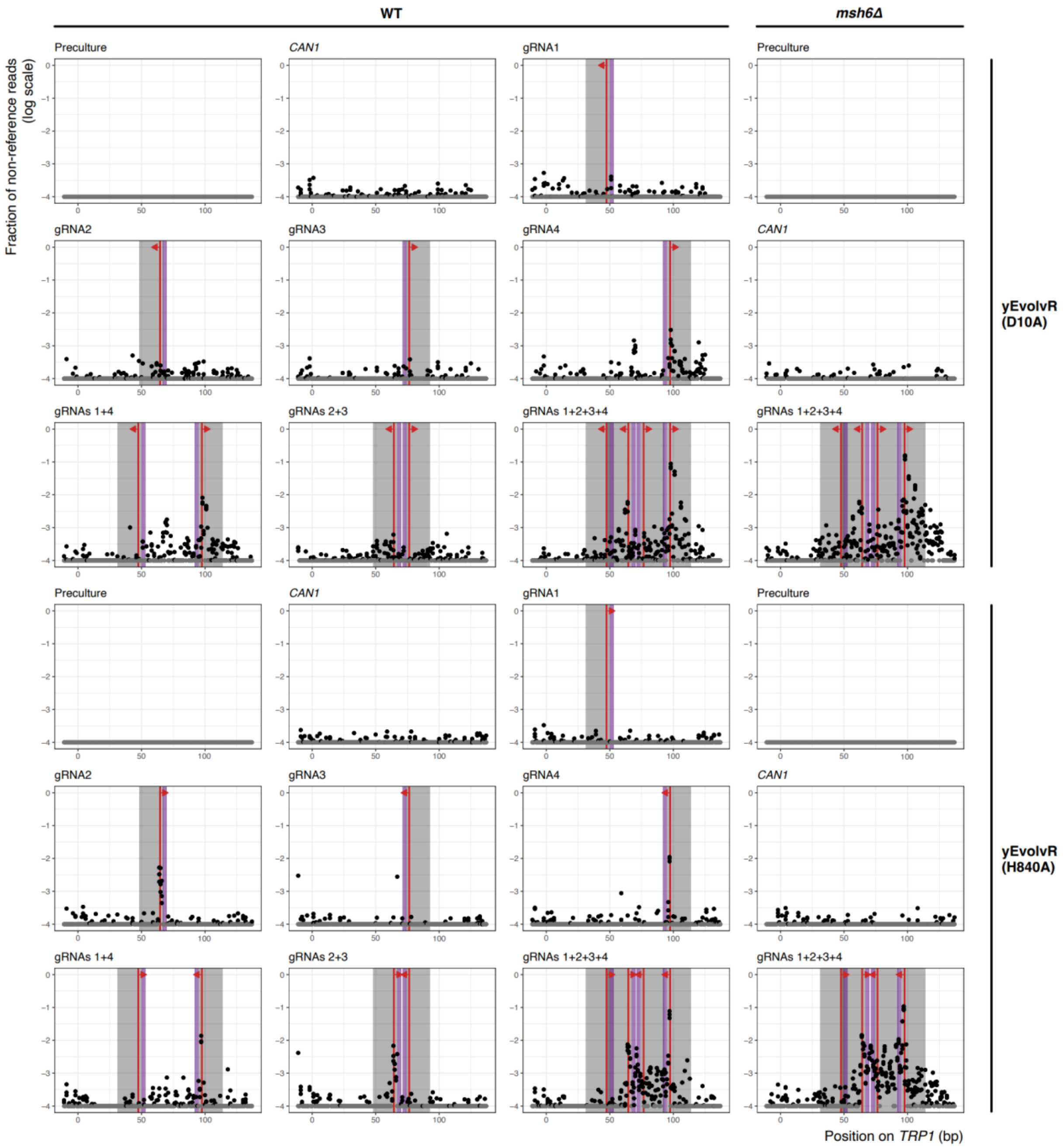

Supplementary Figure S1. Detailed results of amplicon sequencing for individual gRNAs. Mutation frequency as function of position in the gene. Yeast cells were transformed with plasmids encoding yEvolvR, *csy4* and gRNA(s). Liquid minimal medium containing tryptophan was directly inoculated with the transformants. After 72 h of growth, cells were harvested, genomic DNA was isolated and the *trp1* locus was amplified. Cells from the preculture used for transformation served as a control (preculture). Grey box: Target sequence, purple box: protospacer adjacent motif, red line: nick site, red arrow: direction of yEvolvR activity.

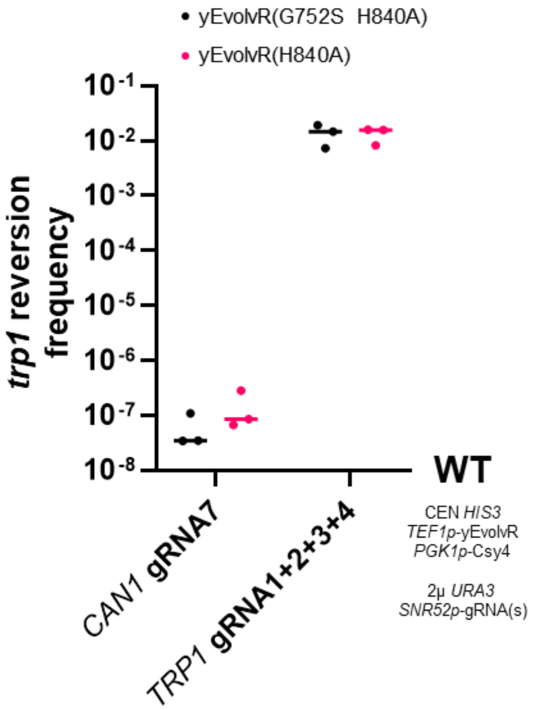

Supplementary Figure S2. *yEvolvR* (H840A) and *yEvolvR* (G752S H840A) generate comparable on-target and off-target *trp1* reversion frequencies. Yeast cells were transformed with plasmids encoding the indicated yEvolvR variant, *csy4* and gRNA(s), and liquid minimal medium containing tryptophan was directly inoculated with the transformants. After 72 h, *trp1* reversion frequencies were determined. Data points are biological triplicates. The bar indicates the median of *trp1* reversion frequencies.
